# The ugliest woman un the world

**DOI:** 10.1192/j.eurpsy.2021.1626

**Published:** 2021-08-13

**Authors:** M. Queipo De Llano, C. Capella Meseguer, E. Rodríguez Vázquez, I. Santos Carrasco, J. Gonçalves Cerejeira, G. Guerra Valera, A. Gonzaga Ramírez, C. Vallecillo Adame, C. De Andrés Lobo, T. Jiménez Aparicio

**Affiliations:** 1 Psichiatry, hospital clinico universitario de Valladolid, VALLADOLID, Spain; 2 Psichiatry, hospital clinico universitario de Valladolid, Valladolid, Spain

**Keywords:** Dysmorphic disorder, Obsessive ideation, autolytic ideation, Depression

## Abstract

**Introduction:**

The main feature of body dysmorphic disorder (BDD) is impairing preoccupation with a physical defect that appears slight or non-existent to others.

**Objectives:**

To draw an overview of BDD through a clinical case of a patient with BDD and autolytic ideation, which improved after an adequate diagnosis and an early pharmacological and psychotherapeutic approach.

**Methods:**

Bibliographic review of the treatment and diagnosis of BDD, from articles published in the last 5 years in Pubmed.

**Results:**

18-year-old woman diagnosed with depression and obsessive ideation, which started at the age of 11, after a comment at school. The patient believes that she has intenseunder-eye bags or dark circles, this has caused her to abandon all activity and self-isolate at home. Symptoms included recurring obsessive and intruding thoughts related to the supposed defect, ritualized behaviors of hours of duration aiming toit through makeup, and autolytic ideation. Therapeutic approach combined psychopharmacological and psychotherapeutic treatments, obtaining gradual improvement of symptomatology and disappearance of the autolytic ideation.

**Conclusions:**

The disorder is severe, which is reflected in high rates of suicide attempts. Differential diagnosis between obsessive and delirious dysmorphophobia is essential for improving outcomes; the egodystonic nature of the symptom, awareness of illness and obsessive personality traits facilitate the diagnosis. A multidisciplinary approach involving psychiatrists and clinical psychologists is necessary for a correct diagnosis and early treatment of this pathology, as well as recognition by dermatologists, surgeons and medical aesthetic professionals, where these patients go with the aim of finding solutions to their problem.

**Disclosure:**

No significant relationships.

